# The ideal healthcare: priorities of people with chronic conditions and their carers

**DOI:** 10.1186/s12913-015-1215-3

**Published:** 2015-12-14

**Authors:** Adem Sav, Sara S. McMillan, Fiona Kelly, Michelle A. King, Jennifer A. Whitty, Elizabeth Kendall, Amanda J. Wheeler

**Affiliations:** Faculty of Health Sciences, School of Allied Health (Public Health), Australian Catholic University, Banyo, Queensland Australia; School of Human Services and Social Work, Menzies Health Institute Queensland, Griffith University, University Drive, Meadowbrook, Queensland Australia; School of Pharmacy, Menzies Health Institute Queensland, Griffith University, Gold Coast Campus, Parklands Drive, Southport, Queensland Australia; School of Pharmacy, Faculty of Medical and Health Sciences, University of Auckland, Auckland, New Zealand; School of Pharmacy, The University of Queensland, 20 Cornwall Street, Woolloongabba, Queensland Australia

**Keywords:** Chronic disease, Unpaid carer, Nominal group technique, Healthcare quality, Australia

## Abstract

**Background:**

It is well established that health consumer opinions should be considered in the design, delivery, and evaluation of health services. However, the opinions of people with chronic conditions and their carers and what they actually consider as ideal healthcare is limited. The aim of this study is to investigate the healthcare priorities of consumers with chronic conditions and their carers, if there are differences between these two groups, and if priorities differ depending on geographical location.

**Methods:**

The nominal group technique was used as a method to identify what is currently important to, or valued by, participants. This method was also particularly suited to learning about healthcare problems and generating important solutions, thereby helping to bridge the gap between research and policy. Recruitment was carried out via purposive sampling, with the assistance of community pharmacies, general practices, various health agencies, government and non-government organisations. A total of 11 nominal groups were conducted; five groups consisted predominantly of consumers (*n* = 33 participants), two groups consisted predominantly of carers (*n* = 12 participants) and four were mixed groups, i.e. consumers, carers, and both (*n* = 26 participants).

**Results:**

The findings suggested that to create a model of ideal healthcare for people with chronic conditions and their carers, appropriate and timely healthcare access was of paramount importance. Continuity and coordinated care, patient-centred care and affordability were equally the second most important healthcare priorities for all groups. When compared with other groups, access was discussed more frequently among participants residing in the rural area of Mount Isa. Compared to consumers, carers also discussed priorities that were more reminiscent with their caring roles, such as increased access and continuity and coordinated care.

**Conclusions:**

Access to healthcare is the most important priority for people with chronic conditions and their carers. In the event of inappropriate access for certain groups, all other efforts to increase the quality of healthcare delivery, e.g. patient-centred care, may be pointless. However, health professionals alone may be limited in their ability to address the concerns related to healthcare access; structural changes by health policy makers may be needed.

## Background

Responding to consumer expectations is one of the criteria that the World Health Organisation (WHO) uses to evaluate health system performance [1]. An understanding not only of what consumers want from their health system (alongside their needs), but also of the relative priorities they place on these expectations is necessary to deliver responsive healthcare. It is accepted that health consumer opinions should be considered in the design, delivery, and evaluation of health services and in creating the conditions that support healthy living [2]. Furthermore, it is recognised that in the absence of appropriate guidance and consumer input, health services can be poorly designed, inefficient and costly [3].

The increasing prevalence of chronic conditions further emphasises the need for health professionals, researchers and policy makers to understand consumer health priorities. People with chronic conditions are relatively high users of health services, and the delivery of optimal services is an important aspect of the management of chronic conditions. Recent research explored the experiences of people with specific chronic conditions and carers, the associated challenges within the health system and suggestions to overcome them [4]. Issues aligned with poor communication or advice, time burden and with medication management [4, 5], findings which were re-enforced by a different project involving people with a range of chronic conditions and their carers [6]. However, while these findings provide important insight into the Australian healthcare system and health systems worldwide, research into what people with chronic conditions and their carers actually consider is *ideal healthcare* is limited [7].

The importance of patient-centred health services has also been emphasised; the ‘needs, wants and preferences of patients and carers should be found and addressed’ [4]. Patient-centred care is recognised as an important requirement of safe and high quality healthcare [8], and one of the most commonly used definitions is: ‘providing care that is respectful of and responsive to individual patient preferences, needs, and values, and ensuring that patient values guide all clinical decisions’ [9]. Thus, the provision of patient-centred care depends on feedback from consumers and carers, as well as health professionals understanding their individual needs and expectations [10]. Although health professionals can ask people directly about their healthcare preferences, the increasing pressures and demand for their skills can prevent them from doing so, e.g. lack of time [11]. Therefore, research is needed to increase our understanding of the ideal characteristics of healthcare services for a diverse range of people with chronic conditions and their carers.

Comparing the needs of consumers and their carers is also important to see if different healthcare priorities arise. Unpaid carers play a valuable role in our society and can have similar, if not more, contact with the health system through providing assistance to the care-recipient. Unpaid carers refer to people who are not employed for their caring role, e.g. a family member. Unpaid carers may also have different priorities if they personally experience a chronic condition. Locality can also have a profound impact on health, as rural dwellers can have unique healthcare experiences compared to their urban counterparts [12]. For example, geographical isolation can bring challenges in terms of limited access to health services and isolation from support networks, particularly in Australia [13, 14]. Thus, the involvement of both unpaid carers and rural dwellers at the interface of healthcare delivery is of paramount importance.

The aim of this study is to investigate the healthcare priorities of consumers with chronic conditions and their carers, if there are differences between these two groups, and if priorities differ depending on geographical location. Given the lack of agreement in the international literature about the best term to describe people with chronic health conditions, we chose the term ‘consumers’, rather than ‘patients’ throughout this study. Unlike consumers, we believe that the term ‘patient’ can be disempowering.

## Method

### Study design

The nominal group technique [15] was used as a method to identify what is currently important to, or valued by, participants, which is the key study objective. The nominal group was developed by Delbecq and Van de Ven as a qualitative research technique for ‘identifying strategic problems and developing appropriate and innovative programs to solve them.’ [15]. The nominal group technique facilities the generation of ideas to problems and/or solutions and asks respondents to rank them (i.e. allocate scores) in order of priority [16]. It also allows the comparisons of such priorities between different groups of participants [17]. This method is particularly suited to learning about healthcare problems and can generate important solutions, thereby helping to bridge the gap between research and policy [18]. The major advantage of this highly structured method over general focus groups is the ability to avoid the group discussion being driven by one or two outspoken individuals; all participants have an opportunity to express their personal priorities [17]. This way, a more in-depth and balanced understanding of consumers’ and carers’ healthcare priorities can be obtained (Table 1).

**Table 1:** The nominal group process

Introduction	The purpose of the study and nominal group process was explained, and the value of each participant’s opinion(s) was reinforced.
Silent generation	Participants were encouraged to record, in silence, as many broad or specific ideas as possible in five minutes, to the following research question: *Imagine an ideal healthcare service several years into the future, what should this service look like?* The aim of this open ended question was to allow participants to think in-depth, about the healthcare they wanted in the future. Participants who had difficulty writing were encouraged to silently think about their answers, or a facilitator assisted them with writing their ideas down.
Round robin	Participant ideas were elicited in a round robin fashion, i.e. everyone had an opportunity to contribute one idea at a time, until all ideas were exhausted. One researcher recorded the ideas verbatim on a whiteboard in front of all participants. Some new ideas were also generated during this process; however, discussion was kept to a minimum to ensure each person felt comfortable to share their idea.
Clarification	In this phase, the group was asked to clarify ambiguous ideas to ensure that the meaning was clearly understood by all participants. The group facilitators remained neutral to the group’s discussion. Similar ideas were then grouped together if there was consensus, and a letter was allocated to each idea for ranking purposes.
Ranking	Participants were asked to individually select their top five ideas, and then rank them in terms of priority, with five marks allocated to their top, and one mark to their lowest, priority.
Discussion	Individual votes for the group were collated for feedback purposes, thus allowing participants the opportunity to discuss their priorities as a group. This final procedure ensured face validity of the healthcare priorities.

Two pilots were conducted to obtain feedback on the content, process and timing of the nominal group technique. Ethical approval was obtained from a University Human Ethics Committee (PHM/12/11/HREC) and written consent received from each participant. Because this study was part of a larger project [19], the number of nominal groups undertaken was dependent on participant time and scope of the project.

### Recruitment

Recruitment was carried out via purposive sampling, with the assistance of community pharmacies, general practices, various health agencies, government and non-government organisations. Nominal group participants were also asked to suggest other people they knew who would be willing to take part in the study, i.e. snowball sampling. Participants were recruited from four Australian regions: the metropolitan areas of Logan-Beaudesert (Queensland) and Perth (Western Australia), and the rural and semi-rural regions of Mount Isa (Queensland) and Northern Rivers (New South Wales).

Participants were included if they self-reported as having a chronic condition themselves, or were caring for someone that did, or both. To account for potential differences in experiences, people who were recently diagnosed with a chronic condition, as well as those with well-established conditions, were included. Participants from culturally and linguistically diverse populations (CALD) and Aboriginal or Torres Strait Islander peoples (IND), were also recruited. People could not participate if they did not meet the above requirements, were under 16 years of age, or did not reside in one of the four data collection sites. Re-imbursement for time was provided in the form of $50 (AUD) supermarket vouchers.

With respect to classifying nominal groups as a consumer, carer or mixed group, the four researchers reviewed the types of participants in each group. It should be noted that a carer group did not necessarily mean that the group involved solely carers; some carers may also personally have had a chronic condition. Mixed groups involved those groups with a mixture of carers, consumers or both, i.e. consumer and carer.

### Procedure

Nominal groups were conducted between December 2012 and April 2013. Participants were asked to: imagine their local ideal healthcare services several years into the future: what services could they offer to help them to meet their individual health goals, or to best support them in their role as a carer? The phrasing of this question built on an appreciative inquiry approach [20], which was designed to enable participants to adopt a positive outlook and think beyond fixing problems and into the future. Three researchers from varying professional backgrounds (e.g. public health and pharmacy, as well as a consumer researcher) facilitated the group process. Each nominal group adhered to the structured process in Table 1, to obtain and compare opinions and priorities between consumers and their carers. All nominal groups were audiotaped and transcribed to better understand the ranked priorities. For each group, Microsoft Office® Excel (v14) spread sheets were used to record participant scores, i.e. the ranked priorities, for each idea listed in the clarification stage (Table 1). This enabled the researchers to identify what the top five priorities were for each group.

### Data analysis

The nominal groups generated two forms of data: *(a)* a quantitative list of individually ranked healthcare priorities, and *(b*) qualitative discussions from transcripts which provided contextual information about the rationale behind priority selection. In-depth analysis was completed in four inter-related stages, i.e. beyond identifying the top five priorities at the conclusion of each nominal group (Table 1).

i) First, individual group scores, i.e. the top five priorities, were emailed to the entire research team to aid continual discussion.
ii) Due to the large number of priorities (*n* = 83) generated from all groups, two researchers from pharmacy and public health backgrounds undertook thematic analysis by grouping similar priorities to develop an analysis framework. Two other researchers independently assessed all priorities to ensure reliability of the analysis. All four researchers then came together to discuss the analysis and agree on a common framework, which resulted in 23 themes [17].
iii) The 23 themes were then presented to the entire research team. As a result of in-depth discussions and further scrutiny of the data, 12 higher order themes were identified. For example, some of the original themes became sub-themes of the 12 higher order themes. For example, respectful, holistic, individualised, empowering, gender specific and cultural awareness, became sub-themes of ‘patient-centred care.’ All ideas listed in the Microsoft Office® Excel (v14) spread sheets (see the above procedure) for each nominal group were then thematically analysed according to the 12 higher order themes. This process was individually undertaken by each of the four researchers, with a discussion held to address any disparities. To further comprehend the 12 higher order themes and solidify the explanation, nominal group transcripts were also analysed by the four researchers using NVivo 9^©^ and the constant comparison method. A consumer researcher, who possessed the necessary life skills and experience, randomly assessed the accuracy of this qualitative analysis.
iv) Finally, for each nominal group, the scores (votes) for each of the 12 higher order themes received were then calculated. Overall analysis of several groups followed the steps as described by van Breda [21]; these results are presented in this paper. Further detailed explanation and exemplar of the entire analytical process used in this study is described elsewhere [17].

## Results

### Study participants

A total of 11 nominal groups (*n* = 71 participants) were conducted; five groups consisted predominantly of consumers (*n* = 33 participants), two groups consisted predominantly of carers (*n* = 12 participants) and four were mixed groups, i.e. consumers, carers and both (*n* = 26 participants). The median number of participants in each group was 6 (minimum = 4 and maximum = 10) and the mean age of participants was 57.6 years across all groups. Overall, there were more females (*n* = 47) than males (*n* = 24), eight participants who identified as Aboriginal or Torres Strait Islander peoples and 24 from Culturally and Linguistically Diverse backgrounds (e.g., Middle Eastern, African, New Zealand/Pacific Islander, Eastern European, etc.). There were 25 participants from the metropolitan areas of Logan-Beaudesert, 11 from Perth, and 9 and 26 from the rural and semi-rural regions of Mount Isa, and Northern Rivers, respectively. Table 2 outlines participant characteristics (ethnicity and gender) by group type (i.e. consumer, carer or mixed) and location.

**Table 2:** Characteristics of participants by group

Location	Group Characteristics						
	Group Type	N	M	F	C	CALD	IND
Logan-Beaudesert	Consumers	6	4	2	6	-	-
	Carers	7	1	6	4	3	-
	Mixed	6	-	6	3	3	-
		6	4	2	1	5	-
Total		25	9	16	14	11	0
Mt Isa	Consumers	4	1	3	-	4	-
	Carers	5	-	5	4	1	-
Total		9	1	8	4	5	-
Northern Rivers	Consumers	8	2	6	-	-	8
		10	6	4	8	2	-
	Mixed	8	3	5	7	1	-
Total		26	11	15	15	3	8
Perth	Consumers	5	1	4	3	2	-
	Mixed	6	2	4	3	3	-
Total		11	3	8	6	5	0
Overall Total		71	24	47	39	24	8

*n* number of participants; *M* Male; *F* Female; *C* Caucasian; *CALD* Culturally and Linguistically Diverse people; *IND* Aboriginal and Torres Strait Islander peoples (Indigenous)

### Ideal healthcare priorities: overall group analysis

To create a model of ideal healthcare for people with chronic conditions and their carers, appropriate and timely healthcare access was of paramount importance. Continuity and coordinated care, patient-centred care, and affordability were equally the second most important healthcare priorities for all groups when the results were combined (Table 3).

**Table 3:** Comparison of top five priorities for participant groups

Consumers (*n* = 5 groups)		Carers (*n* = 2 groups)		Mixed (*n* = 4 groups)		All groups combined (*n* = 11groups)	
Priority	Final Rank	Priority	Final Rank	Priority	Final Rank	Priority	Final Rank
1. Patient-centred care	31.00	1. Access	34.00	1. Access	29.00	1. Access	33.00
2. Access	30.00	2. Continuity and coordinated care	31.00	2. Carer-related issues	28.50	2. Continuity and coordinated care	30.00
3. Affordability	29.50	3. Patient-centred care	28.50	3. Patient-centred care	27.50	2. Patient-centred care	30.00
3. Continuity and coordinated care	29.50	4. Affordability	28.00	4. Continuity and coordinated care	27.00	2. Affordability	30.00
4. Education and information	24.00	5. Carer related issues	25.50	5. Affordability	23.00	3. Quality of service delivery	20.50
5. Quality of service delivery	21.00					4. Education and information	20.00
						5. Legislative changes	18.00

#### Access

Access to healthcare was the most important healthcare priority when group results were combined (Table 3). In particular, it was the highest priority for the nominal groups in Mount Isa and the greater Perth area (Table 4). Access was discussed in the context of physical access, i.e. being able to access healthcare and treatment without delay and in reasonable time; environmental access, i.e. parking spaces at healthcare centres; and to a lesser extent, social access, i.e. equitable healthcare for people from all socioeconomic backgrounds. The difficulties experienced by some participants when accessing healthcare is explicit in the following quote:

**Table 4:** Comparison of top five priorities per location

Location and number of groups	Top five priorities	Final Rank	Location and number of groups	Top five priorities	Final Rank
Logan-Beaudesert (urban - *n* = 4)	1. Continuity and coordinated care	32.50	Northern Rivers (semi-rural *n* = 3)	1. Patient-centred care	35.00
	2. Affordability	31.00		2. Continuity and coordinated care	31.00
	2. Access	31.00		3. Affordability	25.00
	3. Patient-centred care	27.00		4. Carer related issues	24.50
	4. Quality of service delivery	21.50		5. Health promotion	22.50
	5. Legislative changes	18.50			
Mt Isa (rural - *n* = 2)	1. Access	36.00	Perth (urban - *n* = 2)	1. Access	32.00
	2. Affordability	32.00		2. Continuity and coordinated care	31.00
	3. Continuity and coordinated care	29.00		3. Education and information	27.00
	4. Quality of service delivery	28.00		4. Legislative changes	26.00
	5. Legislative changes	21.50		5. Affordability	25.00

*I just would like doctors to be more readily available. You often phone a doctor now when you're sick and they say, sorry, we're fully booked and can't get you in’till next week.* Carer_1013 (Group 13; Perth)

Given that most people had experienced such difficulties and delays with timely healthcare access, they wanted health professionals to be more respectful of, and to acknowledge, their time:

*Now being a full time worker you're taking time off so it's almost like the patients time doesn't matter, but my time is precious.* Consumer and Carer_1049 (Group 7; Logan-Beaudesert)

#### Continuity and coordinated care

Effective communication and collaboration between health professionals, consistency in the messages provided to people, streamlined access to medical histories, and having the same health professionals involved in a person’s care were highly valued:

*I like the idea of having easy access to your medical records. And if you have to transfer those records to another GP [General Practitioner], it should just be done no hassles, no charging [money], no holding you for ransom, just do it.* Carer_1071 (Group 5; Logan-Beaudesert)

In the event of disjointed care, one consumer frustratingly added:

*It comes back to [us] explaining things over again about…your personal health and everything else. *Consumer_1127 (Group 9; Perth)

*Well it's a bit annoying when you're going - so what's your history? I've been coming to you for 20 years. You know? You should have those records. *Consumer_1119 (Group 9; Perth)

A one-stop health centre, where all health professionals and services are co-located, and improved communication and collaboration between health professionals and services, were also important elements of continuity and coordinated care. Possible home follow ups, particularly after hospital discharge, was deemed particularly important:

*…A lady was kicked out of hospital at one o'clock in the morning, who lives alone. So she was put in a taxi to go home alone. And they* [hospital staff] *did no follow up, no nothing…I just think there should be some kind of follow up to make sure that she was okay.* Consumer/Carer_1206 (Group 9; Perth)

#### Patient-centred care

Patient-centred care was more important for consumer groups than mixed or carer groups. The three groups in Northern Rivers also believed that patient-centred care was the most important priority, followed by continuity and coordinated care (Table 4). Participants wanted health professionals to be attentive and listen, and personally know them and their living circumstances. One consumer and carer with a health professional background commented:

*I'd like to go into a place and be treated equally to everybody else and a sense of feeling welcome and knowing someone in there is going to listen and not just treat you as a number.* Consumer and Carer_1143 (Group 4; Logan-Beaudesert)

The importance of healthcare that was gender and culture appropriate, individualised, respectful, empowering and holistic was emphasised:

*I think it's great when…they [medical specialist] actually focus on the holistic view and not just your diabetes.* Consumer_1005 (Group 6; Logan-Beaudesert)

*Putting the care back into care.* Consumer_1115 (Group 8; Northern Rivers)

#### Affordability

Many participants complained about the financial burden of living with and treating their condition(s). The cost of medication, health professional consultations and other health services were discussed extensively, and were often seen to contribute to treatment burden:

*Maybe they* [pharmacies] *could do a payment scheme because a lot of people are not getting their medication because they can’t afford it.* Consumer and Carer_1184 (Group 2; Logan-Beaudesert)

*They* [pharmacies] *have got the price on the prescriptions* [medication label] *now days- the real price, its $900 a month for his* [son] *formula, $200 a month for his tubes, that’s $1100 a month in theory, just to feed him, that’s just one expense. It’s just crazy.* Consumer and Carer_1209 (Group 15; Northern Rivers)

Participants wanted more affordable treatment options, including free or low cost medication, health professional consultations and private health insurance.

#### Quality of service delivery

Quality of service delivery was the third most important priority for all groups when results were combined. This incorporated up-to-date equipment in hospitals, to health professional competency. Participants emphasised the importance of having a qualified and competent heath professional taking care of their treatment. Concepts such as, improving professional standards, duty of care, ethical obligations, confidentiality and understanding, training and development were regularly discussed. Quality of service delivery was also discussed in the context of safe and correct treatment options, with some participants questioning what was recommended to them by their health professionals. Another aspect of quality of service delivery included up-to-date hospital equipment, an issue predominantly raised in rural and semi-rural areas:

*…better equipped hospitals. Most of the equipment in the hospitals now is either way out dated or poorly maintained. It's atrocious.* Consumer and Carer_1018 (Group 15; Northern Rivers)

#### Education and information

Education and information emerged as the fourth most important priority when all groups were combined. However, it was only prioritised by consumer groups and those located in Perth (Table 4). For consumers, education and information was discussed in the context of what information should be provided, by whom, and how. Consumers prioritised the importance of obtaining advice about different treatment options that were available, different support services to assist with managing chronic conditions, and more information about chronic conditions in general. Information about medication, including what they are used for, how to use them, potential side effects and drug interactions, and updates on new medication or treatment changes were deemed a high priority. The importance of offering choice and an explanation about different treatment options, services and support groups were regularly discussed:

*You can have the best health service in the world, if I don't know about it; it may as well not exist.* Carer_1217 (Group 15; Northern Rivers)

Finally, there was agreement that education and information needed to be straightforward, consumer friendly and without medical jargon.

#### Legislative changes

Although legislative changes did not emerge in the top five priorities of the groups when examined individually; it was the fifth most important priority for all the groups combined. These changes included macro-level, government initiated, policy changes to healthcare services that would improve the experiences of people with chronic conditions and their carers. For example, people with chronic conditions being eligible for government subsidies, regardless of their employment status, a cap of private health insurance to make it more affordable, greater medical research funding and free medical devices were discussed.

### Ideal healthcare priorities: key group differences

When compared with other groups, access was particularly discussed among those participants residing in the rural area of Mount Isa, with both groups voting appropriate and timely access as their most important priority. According to these participants, it was not uncommon to wait almost three weeks to see a GP. Accessing treatment at times required large amounts of time and travel, whereby participants could travel over 900 km from Mount Isa to access health services elsewhere. One solution to this issue was discussed in the form of mandatory rural service for healthcare professionals:

*…all doctors, all professionals, as in medical professionals, should be made to do country service.* Consumer_1004 (Group 16; Mt Isa)

Patient-centred care was particularly important for Aboriginal and Torres Strait Islander peoples and culturally diverse participants. Cultural awareness and competency, such as recognising a person’s cultural and religious beliefs, was highly important to these participants, underscoring the need for respectful care. Patient-centred care seemed to be more important for those residing in Northern Rivers than those in Perth, Logan-Beaudesert and the North West regions.

There were also key differences in the priorities of carers, when compared to consumers involved in the nominal groups. Carers discussed the importance of their role being more thoroughly recognised by health professionals and services, the need to work together with the care recipient and the treating health professional, and for extra support and assistance in their role(s). When discussing the difficulties associated with being a carer in helping the care-recipient, one participant explained that they were unable to access treatment on behalf of their mother, who was too ill to visit the GP:

*I called and made an appointment for my mum… “Oh no, you can't see the doctor”. I said why? “It's against the law”. What law? I'm here just to get the prescription for my mum. “Oh no you can't get prescription”…I said I've been coming here for 17 years.* Carer and Consumer_1133 (Group 7; Logan-Beaudesert)

Carers wanted to see macro-level changes within the health system, i.e. easier access to new medication, different ways to navigate the health system, etc. For example, extra support services were recommended to help carers both financially and mentally. A consumer-carer from a mixed group emphasised the lack of support for carers of people who have declining mental health yet are ‘not sick enough’ to be hospitalised:

*…Mental health carers need more support when they're in crisis. Once your loved one is starting to show symptoms - and we're very used to this - you know that there's a crisis imminent and you talk to the professionals and they say, …well is he threatening his own life or is he threatening someone else? Otherwise, you know, it's up to you. Let's wait until he threatens your life.* Consumer and Carer 1016 (Group 15, Northern Rivers)

This issue was of such concern that it was ranked as the second most important healthcare priority by this particular group. The only other group that ranked the higher-order ‘carer’ theme in their top five priorities was another mixed consumer and carer group from Logan-Beaudesert. The two sole carer groups placed greater emphasis on improved access (Mount Isa) or continuity and coordinated care (Logan-Beaudesert), themes which involve the carer’s role.

## Discussion

This study provides important insights about the healthcare priorities of consumers with a range of chronic conditions and their carers. Overall, group findings suggest that accessible healthcare was the most important priority for participants. Everything rested on the premise that people could adequately access healthcare services for treatment. In the event of inappropriate access for certain groups, all other efforts to increase the quality of healthcare delivery, e.g. patient-centred care, may be pointless. Gulliford et al. [22] argued that access is a multidimensional concept, which included four dimensions: adequate supply of health services, personal, financial and organisational barriers. Organisational barriers include issues such as long waiting lists or times to obtain treatment, consultations, or both, systematic variations in referral from primary to secondary care, and limited availability of particular health services [22]. In our study, group discussions during and after the voting process indicated that organisational barriers were the most prevalent dimension of access; timely access to health professionals and services was important. We recognise that health professionals may be limited in their ability to address the concerns related to access, which may require structural changes by health policy makers. One of these changes can involve implementing stronger financial incentives for health professionals to work in rural and/or lower socioeconomic areas.

Continuity and coordinated care emerged as the second most important priority across all nominal groups combined, supporting the findings of Guthrie et al. [23]. These authors suggested that although continuity can be of little concern to young healthy people with minor or acute problems, it is particularly important for elderly people with chronic conditions, as they are heavy users of the healthcare system [23]. Coordinated and continuity of care was also valued more by participants in urban and semi-rural areas as opposed to a rural location, who seemed more concerned about access and affordable healthcare. In contrast, we suspect that the reverse is true in the other three areas, i.e. that they are less disadvantaged in terms of accessible and affordable healthcare. There is a greater need for coordinated and collaborative care between healthcare services and health professionals, especially for people with co-morbidities, who may visit multiple health services for treatment. Previous research demonstrates that a lack of coordinated care can not only lead to time and travel burden for such people, but also result in contradictory advice about chronic conditions and treatment options [24]. However, like access to healthcare, continuity and coordinated care is a multi-dimensional concept, comprising of several components, such as health partnerships, networking, collaboration, and knowledge transfer [25]. Health professionals may be restricted in their ability to implement all components of coordinated care. Yet, offering only some of the components to specific people may be enough to make a positive contribution to a person’s ability to manage their chronic conditions. The cost-effectiveness of implementing these components, their impact on clinical outcomes, and their method of delivery must also be considered [25].

As patient-centred care is generally built upon forming a relationship with a health professional, an opportunity to develop this relationship is more likely to occur if a person receives treatment by one rather than many different health professionals, i.e. if there is care continuity. Health professionals must recognise the unique values and beliefs of their patients, and discuss treatment options as a team with prospective carers. Efforts need to focus on recognising consumers and their carers beliefs, needs and expectations, and adjusting health services to the individual rather than attempting to change the individual. By implementing patient-centred care, most of the other healthcare priorities, such as education and information, service quality, and carer friendly services, may also be addressed, paving the way towards better healthcare. Patient-centred care was particularly important for Aboriginal and Torres Strait Islander peoples, culturally diverse participants, and for those residing in Northern Rivers than those in Perth, Logan-Beaudesert and the North West regions. We suspect that because participants in the latter regions were more concerned about appropriate and timely access or continuity and coordinate care, patient-centred care was seen as a secondary priority, which could only occur once healthcare services were accessed or care was more streamlined.

Unexpectedly, carer related issues emerged as an important priority for mixed groups involving consumers, carers, and participants who were both. While there was reference to carer needs in the two carer groups, other concerns took priority when ranking ideas: access and continuity and coordinated care. On reflection, these themes are not solely relevant for consumers, particularly when unpaid carers assist the care-receiver to manage their healthcare appointments and associated treatments. Furthermore, some of the carers in the carer groups also had a chronic condition.

Overall, there were discussions around greater recognition of the carer’s own health and the carer role by health professionals and services, i.e. being receptive to carer issues. Furthermore, carers wanted to see Government initiatives or legislative changes to better accommodate their role(s). These priorities can be better understood in the context of a carer’s legal rights under the Australian healthcare system. In addition to their social and economic contribution to society overall, unpaid carers have an immediate role in helping the care recipient manage his/her chronic conditions. Although the contributions of carers have been recognised (e.g., The Carer Recognition Act, 2010), many continue to experience difficulties concerning privacy and confidentiality matters, which inhibit their ability to become a partner in the care-recipients health. To address some of these issues, a recommendation put forward by the carers was the introduction of a formal ‘carer card’, which specifies a person’s caring status and authority. This way, it was argued that carers could present this card to obtain personal information about the health and treatment of the carer-recipient. However, the introduction of a formal carer card needs careful consideration. While this system could indeed help carers with the some of the afore-mentioned barriers of becoming a partner in the care-recipients health, it must allow for extenuating circumstances. For example, some consumers may not want their carers to have access to certain medical information, and a consumer-carer relationship may change. This idea, while good in theory, needs further practical considerations.

### Strengths and limitations

Although our study sample included a diverse range of participants, including minority populations, the findings are based on self-reported data and a purposive sampling technique. Hence, the findings may not be generalisable to the broader population of people with chronic conditions and their carers. It was difficult to organise homogenous groups consisting of solely carers, solely consumers, or participants who were both. This made the comparative process across groups more difficult. However, given the limited information available on analysing large data-sets in the literature, a significant amount of time was spent by the researchers on how to best analyse the groups for comparative purposes.

Although the nominal group technique allowed participants to discuss key priorities, such priorities are likely to change in future with the development of technological advances and amendments to the Australian healthcare system. It would be worthwhile to conduct longitudinal studies into this area to more comprehensively understand if, and how, people’s healthcare priorities change over time. We were also unable to distinguish the healthcare priorities unique to specific chronic conditions. The condensing of themes may have biased the results as presumably different themes may have had numerous ideas encompassed under them. Finally, although the majority of participants from culturally and linguistically diverse backgrounds had been in Australia for lengthy periods, it is possible that their priorities may have been influenced by prior experiences of care in other health care systems. Despite these limitations, the study has several strengths. There were several benefits of using the nominal group technique. First, this method provided an opportunity for participants to feel empowered and motivated to express their healthcare priorities. Second, participants in our study were able to reinforce their experiences by discussing them with others who have experienced similar issues [26]. Third, participants were able to discuss and clarify main issues or ambiguous topics, which enhanced the group’s understanding of ideas. Finally, we were able to articulate the healthcare priorities that can be common across a range of chronic conditions, providing health professionals and policy makers with much needed knowledge. Our findings are more likely to be in tune with everyday practices of health professionals, who usually treat people with a variety of chronic conditions rather than one or two more common ones.

## Conclusion

In an era of increased pressures on health systems worldwide, the goal is to ensure that health services are delivered in an efficient and optimal manner. By using a highly structured nominal group method, the findings provide invaluable insights into what may need to occur in order to reach this goal. The most important change is to ensure appropriate and timely access to healthcare. In its absence, resources used to increase the quality of healthcare delivery may be ill-used.
